# Chemical Characterization and Biological Potential of the Essential Oils from the Flowers of Two *Cannabis sativa* L. Cultivars from Komga, South Africa

**DOI:** 10.3390/molecules31111814

**Published:** 2026-05-25

**Authors:** Anwuli E. Odieka, Ayodeji O. Oriola, Gugulethu M. Miya, Pallab Kar, Opeoluwa O. Oyedeji, Mavuto M. Gondwe, Yiseyon S. Hosu, Thami Madliwa, Adebola O. Oyedeji

**Affiliations:** 1 Department of Chemical and Physical Sciences, Walter Sisulu University, Mthatha 5099, South Africa; aoriola@wsu.ac.za (A.O.O.); aoyedeji@wsu.ac.za (A.O.O.); 2 African Medicinal Flora and Fauna Research Niche Area, Walter Sisulu University, Mthatha 5099, South Africa; pallabkar.bio@gmail.com; 3 Department of Chemistry, University of Fort Hare, Alice 5700, South Africa; ooyedeji@ufh.ac.za; 4 Department of Human Biology, Walter Sisulu University, Mthatha 5099, South Africa; mgondwe@wsu.ac.za; 5 Department of Business Management and Economics, Walter Sisulu University, Mthatha 5099, South Africa; yhosu@wsu.ac.za; 6 Eastern Cape Hemp Producers Association (ECHPA), Komga 4950, South Africa; madliwa.endalweni@gmail.com

**Keywords:** plant VOCs, cannabis floral essential oils, hydro-distillation, GC-MS characterization, antioxidant and anti-inflammatory activities, molecular docking

## Abstract

*Cannabis sativa* L. is a medicinal plant cultivated globally due to its remarkable historical and scientific relevance. Through the consumption of its flowers, also referred to as inflorescences, which contain a high content of cannabinoids, terpenes and polyphenols, the therapeutic properties of *C. sativa* can be harnessed. This study therefore aimed to determine the chemical profile, antioxidant and anti-inflammatory activities of the essential oils (EOs) obtained from the fresh and dried flowers of two *C. sativa* cultivars, Lifter and Cherrywine, grown in Komga, South Africa, to assess which cultivar has greater biological potential. The chemical profiles of the hydro-distilled EOs were analyzed by gas chromatography–mass spectrometry (GC-MS), while the in vitro antioxidant and anti-inflammatory activity of the EOs was analyzed using the DPPH and EAD methods, respectively. The identified constituents from the EOs were molecularly docked against NOX2 and NIK (NF-κB-inducing kinase) protein, which are implicated in oxidative stress. The afforded EOs were yellow (pale and bright yellow) in color with a sweet to mildly sweet aroma description. A total of 51 constituents were identified in both fresh and dry oils from the Lifter cultivar, while the Cherrywine cultivar contained a total of 44 constituents. Eighteen compounds, were found to be the main chemical constituents consistent in the flower EOs of both cultivars, notably, caryophyllene (10.71–19.96%), levo-β-pinene (1.37–13.21%), humulene (5.88–9.77%), caryophyllene oxide (4.32–7.49%), D-limonene (1.40–5.48%), α-pinene (2.22–5.22%), nerolidol (0.63–4.97%), cis-β-ocimene (0.22–4.37%), linalool (1.12–4.28%), selina-3,7(11)-diene (0.15–4.23%), humulene-1,2-epoxide (1.23–3.32%), guaiol (0.17–2.60%), (+)-β-selinene (1.20–2.51%), trans-α-bergamotene (0.68–2.37%), β-ocimene (0.90–2.27%), fenchol exo- (0.15–1.27), terpineol (0.14–1.38%) and α-terpineol (0.19–0.75%). The fresh Lifter flower oil (LFO) showed 50% inhibition at 100 μg/mL, with an IC_50_ of 69.50 ± 4.05 µg/mL against DPPH, suggesting moderate to low radical scavenging activity. The maximum percentage inhibition response of DLFO, CFO and DCFO remained below 50% at all concentrations. The antioxidant activity of fresh LFO may be attributed to its overall chemical composition. The flower oils showed in vitro inhibition of protein denaturation; however, the high standard deviation relative to the mean IC_50_ values limited the ability to rank the samples’ potencies. Further in silico studies on the putative constituents in the Lifter and Cherrywine cultivars revealed β-bisabolene and α-curcumene as potential molecular targets, with binding energy scores of −7.7 and −7.9 kcal/mol, respectively. Thus, the study findings highlight the promising biological importance of *C. sativa* inflorescences in the management of oxidative stress-related conditions. Further studies may investigate the influence of environmental growing conditions on their chemical composition, total ROS analysis, pharmacokinetic properties, and in vivo efficacy against oxidative damage to DNA, proteins and lipids. Evaluating the toxicity of the flower EOs is also recommended.

## 1. Introduction

Volatile organic compounds (VOCs) are chemically diverse and lipophilic compounds with low molecular weights and high vapor pressures [1]. They are present in the organs of plants such as roots, flowers, stems, seeds, fruits and leaves, and they play important ecological roles [1]. Essential oils (EOs), also known as volatile oils, are complex mixtures of volatile organic compounds (mainly terpenes and phenylpropanoids) obtained through distillation and extraction with organic solvents and have a characteristic aroma distinct to their plant source [2,3]. EOs represent the concentrated extract of plant VOCs with characterized biological and ecological roles such as protection, communication, defense, pollination and adaptation [1,4,5,6,7]. The amount of essential oils derived from various plant parts is small (usually 1% or less); hence, they are valued for their aromatic properties [2,4,8]. Recently, the research into EOs is a growing area of research interest due to its versatile applications across industries. Plant EOs are used in the food flavoring, agriculture, healthcare, cosmetics, and perfumery industries [2,4]. Their antiseptic and analgesic properties can also be used in dentistry [9]. Anti-tumor, anti-inflammatory, anti-arthritis, insect-repellent, antioxidant, and phytotoxic activities of plant essential oils have been extensively reported in the literature [10,11,12,13].

Generally, VOCs can be classified into three classes: terpenoids, phenylpropanoids/benzonoids, and fatty acids and amino acid derivatives [1]. The terpenoids form the largest group of VOCs, which are derived from the mevalonic acid (MVA) and the methylerythritol phosphate (MEP) pathways. Lipophilic compounds present in essential oils are classified as monoterpenes, diterpenes and sesquiterpenes, depending on the number of isoprene units, and these molecules cross cellular membranes freely and serve various signaling roles inside the cell [1]. Traditional methods used to obtain plant volatiles include distillation (hydro-distillation) and extraction with organic solvents; however, more recent soft extraction methods, such as headspace methods, have been developed [1]. The suitable analytical technique used to obtain the qualitative profiles of the volatiles and measure quantitative changes in volatile or semi-volatile compounds, is gas chromatography–mass spectrometry (GC-MS) [1,14,15]. Flowers produce complex volatile mixtures that constitute a highly diversified group of signaling molecules, with each flower having a unique fragrance due to the relative quantities and diversity of VOCs, as well as the interactions between these compounds [1]. It has been reported that floral chemical profiles vary in many species, both within and between individuals and populations. This intra- and interspecific variation has led scientists to conduct numerous research studies.

Recently, cannabis (*Cannabis sativa* L.), an ancient plant of historical and scientific importance, belonging to the family Cannabaceae family, has been extensively studied due to its medicinal potential and grown to be used as ingredients in several foods, beverages, fibers, nutritional supplements, personal care products and pharmaceutics [16,17,18]. The chemical profile of *C. sativa* EO is of particular interest due to its organoleptic characteristics, which further influences its utilization and biological activity [19,20,21]. *C. sativa* contains various bioactive compounds providing antioxidant, anti-tumor, anti-inflammatory, antifungal, and antibacterial properties [22]. Volatile constituents—such as terpenes, aldehydes, ketones, esters and sulfur-containing compounds—together with non-volatile taste-active molecules including flavonoids and phenolic compounds, underlie its distinctive aroma and flavor [23]. Genetics and environmental influences, cultivation practices, harvest time, seasonal variations and postharvest processing (drying methods, extraction method and conditions during extraction) significantly influence the accumulation of secondary metabolites, including terpenes, flavonoids, esters, aldehydes, ketones and other volatile constituents, as well as the quantity and quality of the EO obtained [24,25,26,27,28]. The cannabis flower, also known as the bud or female inflorescence, contains more than 100 terpenes and terpenoids, which mostly accumulate in the glandular trichomes [13,29,30]. Recently, the interest in the uses of cannabis flower is increasing because, through its consumption, the therapeutic botanical properties of cannabis have been harnessed, as well as its diverse scents (citrus, lemon, sweet, pungent, woody, earthy and herbal) and various ethnomedicinal benefits including for pain management, anxiety and depression [31,32]. The main therapeutic properties of cannabis are attributed to cannabinoids, such as Δ^9^-tetrahydrocannabinol (Δ^9^-THC) and cannabidiol (CBD). Notwithstanding, increasing evidence supports the relevance of terpenes, including monoterpenes and sesquiterpenes, which contribute to their distinctive aroma and serve as natural defense mechanisms against predators, growth modulation, disease resistance, attraction of pollinators, plant–plant communication, and antimicrobial, antioxidant and anti-inflammatory properties [13,26,33]. Also, the entourage effect, arising from the synergistic interaction between cannabinoids and terpenoids, is believed to enhance the therapeutic efficacy of cannabis-derived products [29].

According to the World Health Organization, trade in medicinal plants, raw herbal materials and herbal pharmaceuticals is increasing by approximately 15% annually [34]. This growing acceptance of herbal therapy results from the notion that natural products are safe, inexpensive and readily available [35]. Research has consistently demonstrated a strong and intricate link between oxidative stress and inflammation, considering the former as a primary cause of the latter [36]. Conventional treatments for these conditions often carry adverse side effects, highlighting the urgent need for alternative therapeutic approaches. Consequently, this study determined the chemical profiles of the EOs from the fresh and dried inflorescences of two *C. sativa* commercial cultivars grown in the Eastern Cape Province of South Africa, using gas chromatography–mass spectrometry (GC-MS). The antioxidant and anti-inflammatory capacity of the floral EOs were assessed in vitro using the DPPH radical scavenging spectrophotometric assay and the albumin denaturation assay, respectively. Furthermore, molecular docking of the phytochemicals present in the EOs was performed against NOX2 and NIK (NF-κβ-inducing kinase) proteins to explore potential molecular targets and mechanisms of action underlying the bioactivities of the studied EOs as antioxidant and anti-inflammatory agents.

## 2. Results and Discussion

### 2.1. Physicochemical Analysis of Fresh and Dried Flower EOs from Two C. sativa Cultivars

The cultivars evaluated were commercially available varieties, and because they are grown under various conditions, the observed differences may result from both genetic and environmental factors rather than solely genetic variation. Fresh and air-dried materials were analyzed as distinct postharvest states, and yields were expressed as a percentage of fresh weight (*w*/*w*), calculated as the mass of the extracted material divided by the mass of the starting material at the time of analysis. The results are shown in Table 1. Upon hydro-distillation, the fresh Lifter flower oil (LFO) and the dried Lifter flower oil (DLFO) afforded yields of 0.64% and 0.91% (*w*/*w*), respectively. On the other hand, the fresh and dried flower oils from the Cherrywine cultivar (CFO and DCFO) had yields of 0.27% and 1.99% (*w*/*w*), respectively. Overall, the DCFO exhibited a higher oil yield. That said, the yield range for the fresh and dried flower oils was 0.05–18.0% (*w*/*w*), which aligns with the literature [37], and the variation in EO yields suggests differences in essential oil content. The EOs obtained were yellow in color and had a mildly floral, sweet smell. This could indicate oils with high concentrations of monoterpenes and sesquiterpenes, characterized by a sweet, floral and woody aroma [23]. Monoterpenes such as ocimene, with a fruity fragrance, and linalool, known for its lavender-like scent, are commonly found in cannabis. Additionally, sesquiterpenes such as bisabolol, characterized by a sweet floral aroma; nerolidol, known for its woody, citrusy scent; and caryophyllene and humulene, associated with an earthy, woody aroma, could contribute to the aroma of the flower oils [23].

### 2.2. Chemical Composition of Floral EOs of Two C. sativa Cultivars

The chemical compositions of the inflorescence (flowers) EOs from two cultivars of *C. sativa*, as revealed by the chromatographic profiles, are presented in Figure S1 and Table 2. The main chemical constituents in the flower EOs of both cultivars include caryophyllene (10.71–19.96%), levo-β-pinene (1.37–13.21%), humulene (5.88–9.77%), caryophyllene oxide (4.32–7.49%), D-limonene (1.40–5.48%), α-pinene (2.22–5.22%), nerolidol (0.63–4.97%), cis-β-ocimene (0.22–4.37%), linalool (1.12–4.28%), selina-3,7(11)-diene (0.15–4.23%), humulene-1,2-epoxide (1.23–3.32%), guaiol (0.17–2.60%), (+)-β-selinene (1.20–2.51%), trans-α-bergamotene (0.68–2.37%), β-ocimene (0.90–2.27%), fenchol exo- (0.15–1.27), terpineol (0.14–1.38%), α-terpineol (0.19–0.75%) and naphthalene,1,2,3,4,4a,5,6,7-octahydro-4a,8-dimethyl-2-(1-methylethenyl) (0.58–0.90%). The flower oils contained predominantly oxygenated sesquiterpenes, except for DLFO, which had almost the same amount of monoterpenes (43.78%) as of sesquiterpenes (46.43%) present. In addition, the fresh plant materials (LFO and CFO) contained more chemical components. Interestingly, some EO constituents were present exclusively in the fresh and dried plant materials.

Notable sesquiterpenes found exclusively in LFO include nerolidol (1.03%), ledol (0.32%), caryophylla-4 (12),8(13)-dien-5-α-ol (0.56%) and bicyclogermacrene (0.68%). cis-β-Ocimene, (Z), fenchol exo-, pinene hydrate, cis-p-mentha-2,8-dien-1-ol and α-terpineol were the monoterpenes present exclusively in the fresh Lifter oil (LFO), whereas monoterpenes such as camphene, β-terpinene (19.23%), β-ocimene, fenchol and trans-2-pinanol were present exclusively in the dried flower oil (DLFO). Nerolidol (0.63%) with a different CAS number was identified, along with other sesquiterpenes, present exclusively in DLFO. Notable sesquiterpenes present exclusively in the Lifter cultivar include ylangene, α-amorphene, valencene, β-bisabolene, alloaromadendrene, γ-cadinene, β-eudesmol, α-eudesmol, bulnesol and α-bisabolol. On the other hand, rosefuran, myroxide, β-ocimene and α-copaene were monoterpene compounds found exclusively in the fresh Cherrywine flower oil (CFO), while sesquiterpenes such as δ-guaiene, 4,11-selinadiene, guaia-9,11-diene, 4,5-di-epi-aristolochene, (E)-β-farnesene and (Z,Z)-α-farnesene were present in CFO. The monoterpenes found exclusively in the dried Cherrywine flower oil (DCFO) are β-pinene (13.21%), trans-β-ocimene, cis-β-ocimene (4.37%) and α-terpineol. Interestingly, the sesquiterpenes found exclusively in the DCFO are isocaryophyllene and patchoulane. Notable compounds found exclusively in both fresh and dried plant parts of the Cherrywine cultivar include caryophylla-4(12),8(13)-dien-5-β-ol, β-sesquiphellandrene, α-selinene and α-curcumene. All terpenes identified in this study are reported in previous studies [20,26,38,39], except for patchoulane, α-curcumene, rosefuran and myroxide, which are not widely reported in *C. sativa* literature.

Although these sesquiterpenes and monoterpenes have been found in other medicinal plants, their biological activities have been reported. For example, patchoulane, the sesquiterpene found exclusively in DCFO, has been reported in Cyperus rotundus (Cyperaceae), which showed moderate cytotoxicity against human ovarian cancer cells [40]. Computational studies of rosefuran, a monoterpenoid-derived furan, suggest its modest antioxidant/radical scavenging capacity in essential oil mixtures [41]. Myroxide, an oxirane derivative of ocimene, is a monoterpene present exclusively in CFO and has been reported to be present in elevated levels in the EO samples of Artemisia absinthium exploited for its antimicrobial, antioxidant and anti-inflammatory properties [42]. α-Curcumene is reported to have antimicrobial, antioxidant, anti-cancer and anti-inflammatory activities [43,44]. Caryophylla-4(12),8(13)-dien-5-β-ol, found in CFO, has been reported in Psidium species, and the fresh stembark of *C. sativa* has cytotoxic, antifungal, anti-inflammatory, larvicidal, antimicrobial and antioxidant properties [45,46]. It is worth noting that the EOs were cannabinoid-free, unlike in previous reports [47]. Most of these natural monoterpenes and sesquiterpenes from the cannabis flower have relaxing sedative and anti-depressive effects. Oxygenated sesquiterpenes such as α-bisabolol, bulnesol and α-eudesmol, present in pharmacologically appreciable quantities in the Lifter flower oils, have been reported to have antifungal, antiplatelet, anticholinesterase, antioxidant, antiemetic, anti-inflammatory, anticancer and anti-tumor activities [13,48,49]. Caryophyllene, caryophyllene oxide and humulene, present in both cultivars, have also been reported as the main constituents of cannabis flower oil [17,33]. Consequently, given previous biological reports on the terpenes of *C. sativa*, the flower EOs of both cultivars studied may be a potential source of therapeutic agents for disease management.

### 2.3. Evaluation of In Vitro Antioxidant Activity of the Fresh and Dried C. sativa Floral EOs of Two C. sativa Cultivars

In this study, the 2,2-diphenyl-1-picrylhydrazyl (DPPH) radical scavenging assay was used. This spectrophotometric method was used to quantify the free radical scavenging activity of the flower EOs of two *C. sativa* cultivars. The spectrophotometric method is sensitive, cost-effective, reproducible, rapid and can measure antioxidant capacity in any extract compared with antioxidant standards such as L-ascorbic acid [50]. The antioxidant activities of the EOs from fresh (LFO, CFO) and dried (DCFO, DLFO) flowers of both C. sativa cultivars were determined as indicated by the 50% inhibitory concentration (IC_50_) of the radicals, as well as the standard (ascorbic acid).

**Figure 1:**
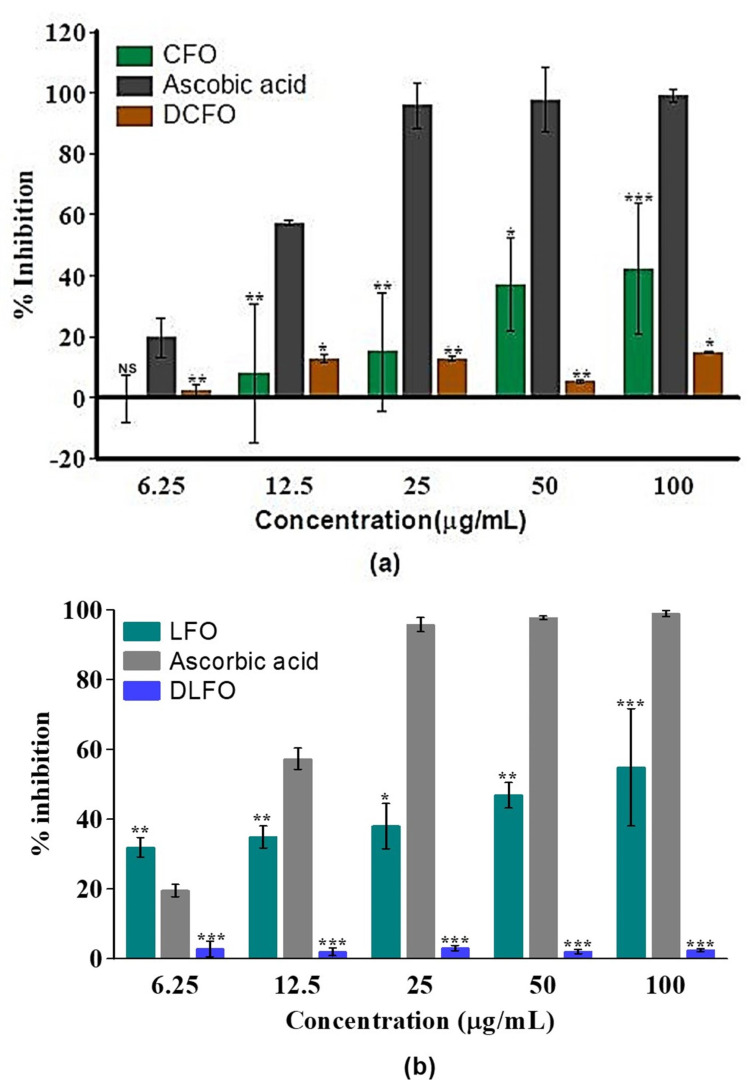
(**a**) In vitro anti-inflammatory activity of fresh (CFO) and dried (DCFO) flower oils by DPPH method. Data are represented as the mean ± S.D. (*n* = 3). * *p* ≤ 0.05; ** *p* ≤ 0.01; *** *p* ≤ 0.001; NS = non-significant when compared with the standard ascorbic acid. (**b**) In vitro antioxidant activity of fresh (LFO) and dried (DLFO) flower oil by DPPH method. Data are presented as the mean ± S.D. (*n* = 3). * *p* ≤ 0.05; ** *p* ≤ 0.01; *** *p* ≤ 0.001 when compared with the standard (ascorbic acid).

The results in Table 3 and Figure 1a,b show that the % inhibition of most flower EOs remained below 50% across all concentrations; hence, the IC_50_ values could not be determined. At a low concentration, fresh Cherrywine flower oil (CFO) showed negative % inhibition values, indicating low scavenging potency. Increased inhibition was observed at higher concentrations, and the compound showed better activity than DCFO. The fresh Lifter flower oil (LFO) showed significantly (*p* < 0.001) higher antioxidant activity at 100 μg/mL, with an IC_50_ value of 69.50 ± 4.05; however, the lower IC_50_ value (11.28 ± 0.20) of the standard (ascorbic acid) indicates that the standard possesses greater antioxidant potency overall. LFO, with levo-β-pinene (12.35%) and caryophyllene (10.71%) as its major constituents, showed better antioxidant activity than DLFO. Studies [51,52] have shown that the major compounds in LFO can scavenge reactive oxygen species, but CFO, DCFO and DLFO contain a higher percentage of caryophyllene. This observation suggests that the antioxidant potency of fresh Lifter flower oil (LFO) is influenced by its overall chemical composition rather than by its major constituents. Notably, LFO contained unique constituents, including pinene hydrate (0.27%), ledol (0.32%), caryophylla-4(12),8(13)-dien-5-α-ol (0.56%), bicyclogermacrene (0.68%), nerolidol (1.03%) and cis-p-mentha-2,8-dien-1-ol (0.16%). Minor constituents at low concentrations can contribute to biological activity, as synergistic interactions between major and minor compounds may enhance overall antioxidant activity. Perhaps the poor dose–response and negative inhibition values observed from some of the tested EOs (CFO, DCFO and DLFO) may be due to subtle chemical compositional changes and possible chemical changes (oxidation and degradation) due to prolonged storage, as cannabis essential oils become chemically unstable over time, which can produce secondary products that can affect the oils’ quality and efficacy [33,38,53]. The antioxidant assay provided reproducible results with the standard control (ascorbic acid), which indicates that the observed sample variability is not a methodological issue. It is worth noting that re-extracting the essential oils to assess the potential impact of prolonged sample storage was not performed in the present study.

### 2.4. Inhibitory Effect of C. sativa Flower Oils on Protein Denaturation

**Figure 2:**
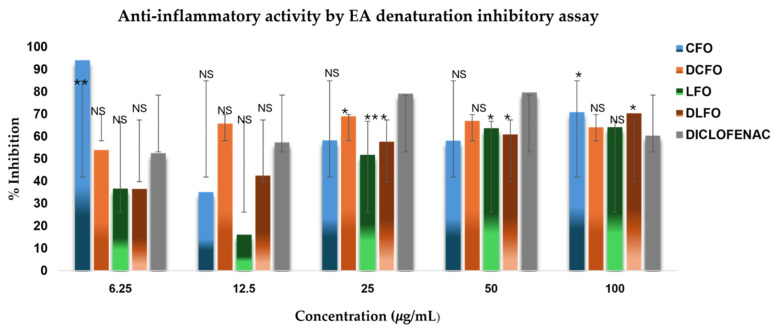
In vitro anti-inflammatory activity of flower oils of two *C. sativa* cultivars. Results are given as the mean ± S.D. (*n* = 3). * *p* ≤ 0.05; ** *p* ≤ 0.01; NS = non-significant.

The protein (egg albumin) denaturation test is an analytical technique for rapidly and reliably assessing the anti-inflammatory activity of natural products [54]. The mean percentage inhibitions and IC_50_ values of the flower EOs from two *C. sativa* cultivars, with egg albumin denaturation, are shown in Figure 2 and Table 4 below. At the lowest concentration of 6.25, CFO exhibited 94.18 ± 0.08 inhibition, which was significantly (*p* ≤ 0.01) higher than that of diclofenac (52.58 ± 4.16). Diclofenac showed greater potency and reproducible inhibitory activity, with an IC_50_ of 9.25 ± 1.09, compared with the flower oils. The flower EOs exhibited higher IC_50_ values and greater variability in their inhibitory response, indicating lower potency than the standard. The fresh LFO exhibited an IC_50_ value of 16.12 ± 10.01, lower than those of DLFO, CFO and DCFO, but with reduced precision due to variability in their inhibitory activity. The EOs from the Cherrywine cultivar had the least potency relative to their IC_50_ values. Overall, the 50% inhibitory concentration (IC_50_) values of the EOs could not be used to rank the potency of the flower oils or the cultivars due to the high standard deviation relative to the mean IC_50_. This variability in inhibitory responses observed in the tested EOs may be due to differences in sample composition and compositional changes that influence bioactivity [55,56]. The positive control (diclofenac) exhibited consistent results across replicates, confirming the reliability of the assay conditions.

### 2.5. In Silico Molecular Docking of Flower Oil Constituents

**Figure 3:**
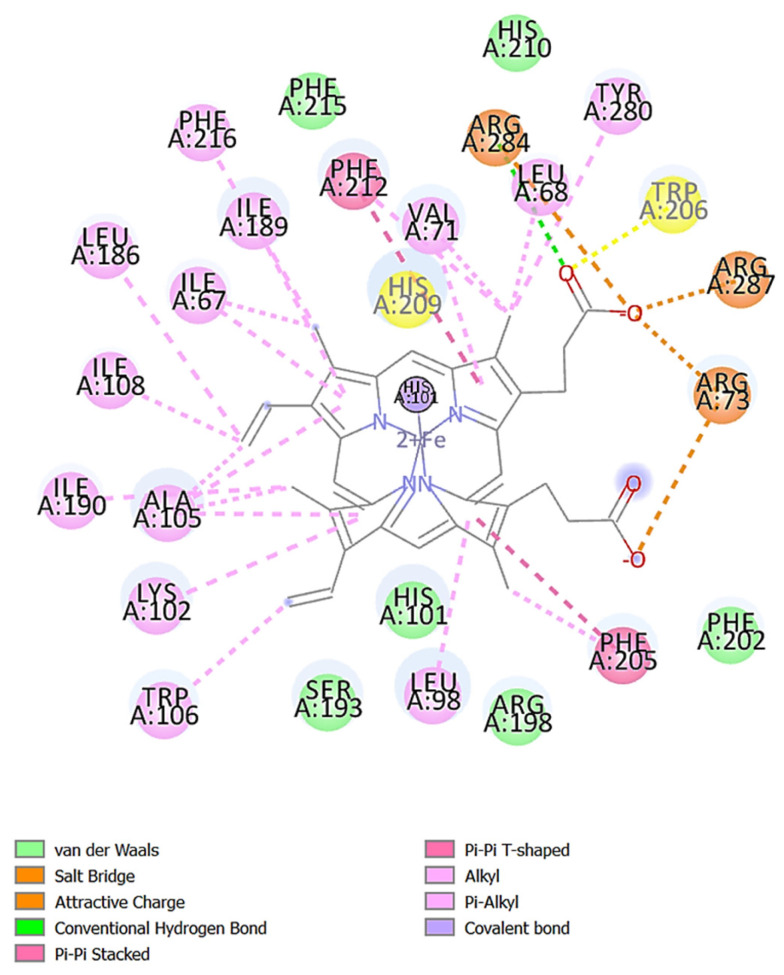
Amino acids forming the active site pocket in NOX2.

**Figure 4:**
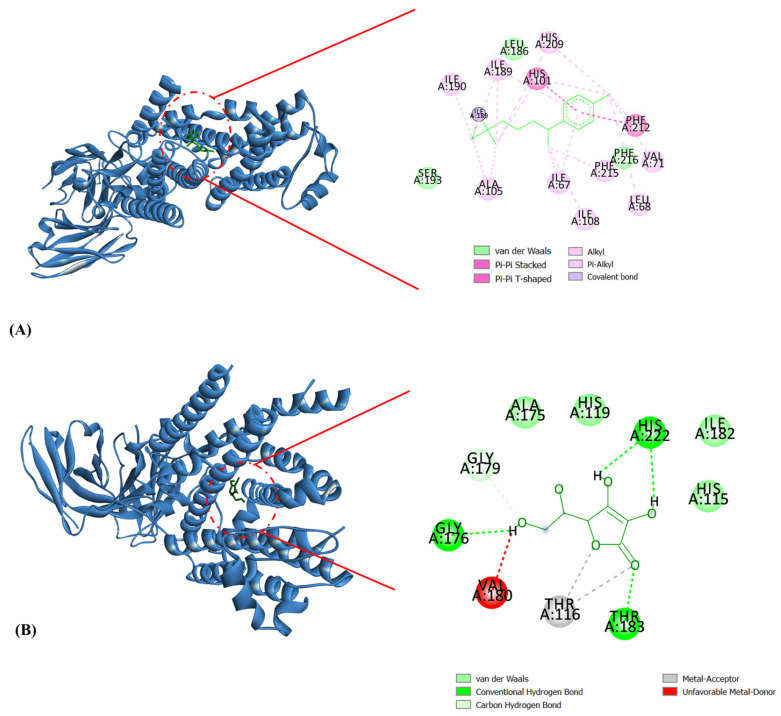
Molecular docking interactions of NOX2 with (**A**) α-curcumene and (**B**) L-ascorbic acid. The blue ribbon represents the NOX2 protein. α-Curcumene and L-ascorbic acid are shown as green sticks.

The NOX2 and NIK (NF-κβ-inducing kinase) proteins were molecularly docked against the main components found in the essential oils from the flowers of two *C. sativa* cultivars. The results of the research showed that Ile67, Leu186, Ile189, Phe216, Phe215, Phe212, His209, His101, Leu68, Val71, Arg284, His210, Tyr280, Trp206, Arg287, Arg73, Phe202, Phe205, Arg198, Leu98, Ser193, Trp106, Lys102, Ala105, Ile190 and Ile108 formed the active site pocket in NOX2 (PDB ID 7U8G) (Figure 3). Table 5 displays the binding energy scores of the EO constituents found exclusively in the dried and fresh flowers of the Cherrywine cultivar against the NOX2 protein. Among the phytocompounds, α-curcumene showed the best binding energy score of −7.5 kcal/mol with hydrophobic interactions (Leu186, Ser193, Phe216), Alkyl and Pi-Alkyl interactions including (Ile67, Leu68, Val71, Ala105, Ile108, Ile189, Ile190, His209, Phe215), Pi–Pi stacked and Pi–Pi T stacked interactions (His101, Phe212) and a covalent bond (Ile189) (Figure 4A). It is interesting to note that these residues, located in the active site (Figure 4), are associated with α-curcumene, a compound shown to play a crucial role in the interaction between NOX2 and small-molecule inhibitors (Figure 4A). The enzyme NOX is a membrane-bound ROS producer. When the enzyme NOX moves an electron from one oxygen atom to another, superoxide is created. CAT uses reduced GSH to catalyze the further conversion of hydrogen peroxide, which it first converts from superoxide to water. The standard L-ascorbic acid showed a binding energy score of −5.7 kcal/mol with hydrophobic interactions (His115, His119, Ala175, Ile182) and hydrogen bonds (Gly176, Gly179, Thr183, His222) (Figure 4B).

**Figure 5:**
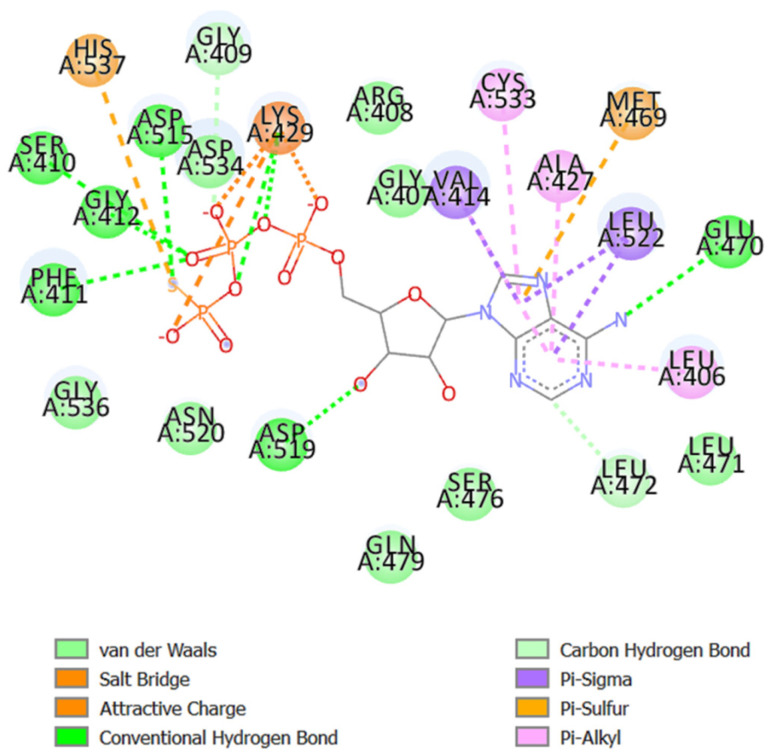
Amino acids forming the active site pocket in NIK.

**Figure 6:**
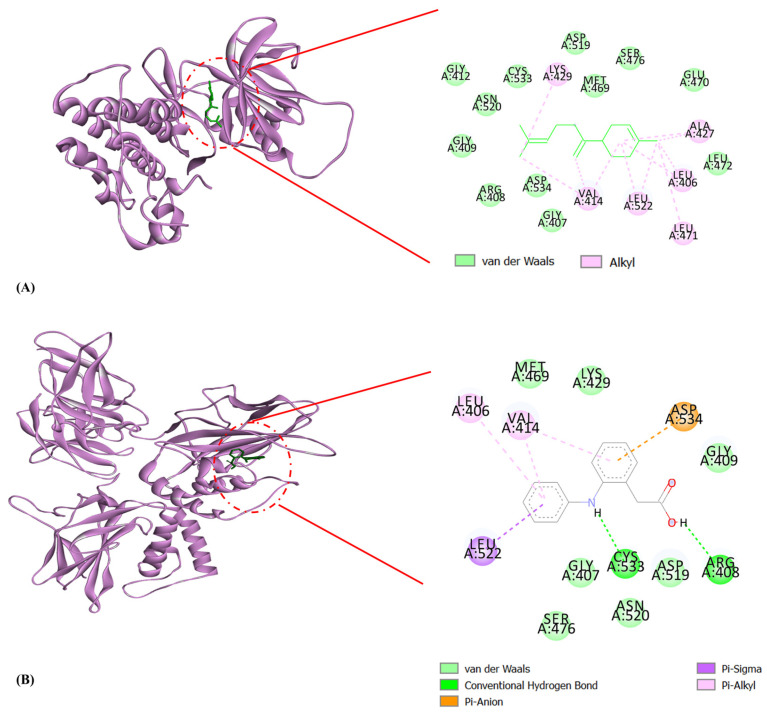
Molecular docking interactions of NIK with (**A**) β-bisabolene and (**B**) diclofenac. The pink ribbon represents the NIK protein. β-bisabolene and diclofenac have been illustrated as green sticks.

The NIK (NF-κβ-inducing kinase) protein and the native ligand produced the active site pocket (Figure 5). Table 6 displays the binding energy scores of the essential oil compounds found exclusively in the dried and fresh flowers of the Lifter cultivar against NIK. The molecule β-bisabolene was found to have a slight upper hand compared with the remaining constituents, returning a binding energy score of −7.7 kcal/mol and interacting with the hydrophobic interaction (Gly407, Arg408, Gly409, Gly412, Met469, Glu470, Leu472, Ser476, Asp519, Asn520, Cys533, Asp534) and alkyl (Leu406, Val414, Ala427, Lys429, Leu471, Leu522) of the protein NIK (Figure 6A). However, the standard diclofenac is not far behind: it showed −5.9 kcal/mol with the hydrophobic interactions (Gly162, Asn164, Leu173, Ala174, Gln177, Phe217, Thr226) and hydrogen bond (Leu167), Pi-Alkyl (Val414, Ala427, Lys429, Met469, Leu522, Cys533) interactions (Figure 6B). Interestingly, Figure 6 shows that these active site residues are associated with β-bisabolene, a compound shown to play a significant role in the interaction between NIK and small-molecule inhibitors (Figure 6A). NIK plays an important role as an upstream regulator of the NF-κβ signaling pathway for inflammation. Therefore, for illnesses associated with inflammation, blocking or inhibiting NIK may be a promising strategy to reduce or modulate inflammatory disease.

## 3. Materials and Methods

### 3.1. Reagents and Experimental Procedure

HPLC-grade solvent (hexane, 2.5 L), DPPH (1,1-diphenyl-2-picrylhydrazyl), fresh eggs, diclofenac, ascorbic acid, glassware, and vials were sourced from Sigma-Aldrich (Pty) Ltd. (Johannesburg, South Africa) through a licensed local supplier, Shalom Laboratories (Musgrave Road, Durban, South Africa). Distilled water was prepared and used for hydro-distillation with a Clevenger-type apparatus. Absorbance values of the DPPH and EAD assays were obtained on a 680-Bio-Rad microplate reader (serial number 14966, Bio-rad Laboratories, Inc., Hercules, CA, USA).

### 3.2. Ethical Consideration

A permit for research on *Cannabis sativa*, including cannabis collection, was obtained from the South African Health Products Regulatory Authority (SAHPRA) under permit No. PIA-HP-EC-2022-0023. Furthermore, approval for the execution of the study was obtained through the Walter Sisulu University research ethics committee, with approval reference number WSU/FNS-GREC-2021/12/01/G9. Fresh whole cannabis plants of the two cultivars studied were also deposited at Walter Sisulu University’s herbarium for authentication, with the voucher numbers AEO-001 and AEO-002 assigned to the Lifter and Cherrywine cultivars, respectively.

### 3.3. Sample Collection and Extraction Procedure

The cultivars were obtained from the Eastern Cape Hemp Producers’ Association (ECHPA) in South Africa. The seeds of the Lifter cultivar with the serial number Lot#:2020 - -A6, were sourced from Jack Hempicine LLC, 3395 S Pacific Hwy. W, Independence, Oregon, USA (OR 97351) and was cultivated using the greenhouse approach, while Cherrywine was sourced from Bodhi Urban of Longmont, Colorado, USA (CO 80501) and was cultivated outdoors. Notably, Cherrywine is a hybrid of 50% indica and 50% sativa, while the Lifter cultivar is 100% sativa. Dried (shed-dried in the dark at 25 °C for 14 days) and fresh flowers of the two *C. sativa* cultivars were obtained in sealed FDA-approved polyethene bags from Komga in the Eastern Cape province of South Africa (GPS coordinates: 32.577°S 27.888°E). Extraction of EOs from fresh and dried flower samples was carried out following ASTM (D8282-19) guidelines [57] and according to the methods of Naz et al. [58] and Oyedeji et al. [59] with slight alterations. A hydro-distillation method using a Clevenger-type apparatus was employed to extract essential oils from the flowers of the cultivars. This technique is reliable, enables efficient isolation of volatile constituents, and the % yield (*w*/*w*) standardizes the results obtained regardless of the amount of plant material available. The fresh flower samples were extracted immediately upon arrival from the farm, while the dried samples were extracted subsequently. A sample-to-solvent ratio sufficient to ensure that the solvent (distilled water) level was above the level of the plant material was used in a round-bottom flask [60]. The round-bottom flask was placed on the heating mantle, and the temperature was set to 100 °C for 1 h, then decreased to 80 °C for 3 h, for a total extraction time of 4 h, until no further EO accumulation was observed, indicating that the extraction was complete. The EO was collected over a hexane–water mixture. The EO accumulated in the hexane portion was collected and stored in glass vials at 0–4 °C until analysis.

### 3.4. Physicochemical Analysis of the EOs

The yields of the EOs were determined gravimetrically (*w*/*w*) as the percentage (%) yield of fresh (LFO, CFO) and dried (DLFO, DCFO) flower of the *C. sativa* cultivars using Equation (1)

(1) $$\mathrm{Essential}\;\mathrm{oil}\;\mathrm{yield}\;(\%)=\frac{W1}{W2}\times 100$$

where W1 = net weight of oils (grams) and W2 = total weight of fresh/dried plant (grams) The oil yield is reported as (% *w*/*w*). Gas chromatography coupled with mass spectrometry (GC-MS) was used to determine the chemical composition (terpene profiles) of the extracted essential oils.

### 3.5. GC-MS Sample Preparation and Analysis

Chromatographic separation was performed on a gas chromatograph (6890 N, Agilent Technologies Inc., Santa Clara, CA, USA) coupled to an inert XL EI/CI Mass Selective Detector (MSD) (5975B, Agilent Technologies Inc., Palo Alto, CA, USA). The GC-MS system was coupled to a CTC Analytics PAL autosampler. Separation of the essential oils was performed on a 5% phenyl dimethylpolysiloxane fused-silica non-polar ZB-5MS (30 m, 0.25 mm ID, 0.25 μm film thickness) capillary column (model number: Zebron 7HG-G010-41, Phenomenex Inc., Torrance, CA, USA). Helium was used as the carrier gas at a flow rate of 1 mL/min. In total, 50 μL of the sample was diluted with 950 μL of acetone before injection into the GC-MS, 1 μL of the EOs of *C. sativa* cultivars was diluted in 4 μL n-hexane, and the mixture was injected on the GC operated at a 50:1 split ratio. The injector temperature was maintained at 240 °C. The oven temperature was programmed as follows: 50 °C for 6 min, ramped at 5 °C/min to 320 °C, and held for 2 min. The MSD was operated in full scan mode, and the source and quad temperatures were maintained at 230 °C and 150 °C, respectively. The transfer line temperature was maintained at 250 °C. The mass spectrometer was operated in electron impact (EI) mode at 70 eV, scanning from 35 to 650 *m*/*z*.

#### Identification and Quantification of the EOs

The chemical compounds were quantified on the basis of the relative response in the MS chromatograms and identified through their linear retention indices (LRI) relative to a series of n-alkanes (C_9_–C_36_) on the same capillary column. The LRI values obtained were compared with values reported in the NIST web book, ChemSpider, PubChem and other literature [59,61,62,63].

### 3.6. Determination of Antioxidant Activity

The DPPH free radical scavenging activity of the EOs was determined following a previously reported method [64] with slight modifications. A total volume of 40 mL of 0.1 mM DPPH free radical in methanol was added to 1 mL (1000 μL) of a serially diluted oil sample at 100, 50, 25, 12.5 and 6.25 μg/mL in triplicate. Next, 100 μL of the test samples and the control solution was transferred to a 96-well plate and incubated in the dark at room temperature for 30 min. The absorbance was measured at 490 nm on a microplate reader. Radical scavenging activity was calculated according to Equation (2).

(2) $$\%\;\mathrm{DPPH}\;\mathrm{inhibition}=\frac{ABScontrol-ABSsample}{ABScontrol}\times 100$$

where ABSsample = absorbance of the test sample, while ABScontrol = absorbance of the negative control (methanol). The data obtained were plotted against the concentration of the oil samples, and the fitting of the data to the dose–response equation provided the IC_50_ values, which show the concentration that caused a 50% inhibition of the DPPH radical.

### 3.7. In Vitro Anti-Inflammatory Analysis of EOs

The in vitro anti-inflammatory potential of the floral EOs was determined by their inhibitory effect on protein denaturation using the egg albumin denaturation assay, as described by Ameena et al. [65], with slight modifications. For this, 0.1 mL of albumin from a fresh chicken egg, 1.4 mL of phosphate-buffered saline with a pH range of 6.8–7.4 (15 M) and 1 mL of *C. sativa* flower oil at concentrations of 6.25, 12.5, 25, 50 and 100 µg/mL were mixed in three replicates. The reaction mixture was incubated at 37 °C for 15 min away from direct light. Then, it was heated to 70 °C and held for 5 min in a thermostatic water bath. The resulting mixture was allowed to cool before the absorbance was measured at 490 nm. Diclofenac was used as the reference anti-inflammatory drug. The percentage inhibitory effect of the EOs on egg albumin denaturation (EAD) was calculated according to Equation (3).

(3) $$\%\;\mathrm{inhibition}\;\mathrm{of}\;\mathrm{EAD}=\frac{A0-A1}{A0}\times 100$$

where EAD = egg albumin denaturation, A0 = absorbance of the control and AI absorbance in the presence of samples and standard. The concentration that inhibited EAD by 50% was taken as the IC_50_ value, which was calculated by linear interpolation between concentrations immediately above and below 50% inhibition.

### 3.8. Statistical Analysis

The antioxidant and anti-inflammatory assay results were analyzed using KyPlot software, version 6.0 (Kyenslab, Tokyo, Japan). Differences between the standard and EO samples were compared for significance using a parametric test (paired *t*-test). All measurements are expressed as the mean ± standard deviation (*n* = 3). Linear square regression was selected to calculate IC_50_ values. Differences considered significant (*p* ≤ 0.05) are labeled with an asterisk (*) within the bar graphs.

### 3.9. In Silico Molecular Docking

Molecular docking analysis was conducted using the main chemical constituents of the essential oils from the flowers of two *C. sativa* cultivars to investigate their potential to mitigate oxidative stress and inflammation. The Protein Data Bank (http://www.rcsb.org) provided the PDB structures of NOX2 and NIK (NF-κB-inducing kinase), with the PDB IDs 7U8G [66] and 4DN5 [67], respectively. After removing the water atoms from the protein structures, AutoDockTools (version 1.5.6) were used to add polar hydrogen atoms and Kollman charges in preparation for docking. The NCBI PubChem (https://pubchem.ncbi.nlm.nih.gov/) was used to download the phyto-compounds. The downloaded SDF structures were then converted to PDB format using the Open Babel Server [68]. The ligand structures were optimized for energy using the PRODRG server [69], and then their energies were subsequently minimized using the GROMOS 96 force field. The Discovery Studio visualization program was used to visualize the docked complexes after Autodock Vina completed the molecular docking process [70,71].

## 4. Conclusions

This study investigated the flower (inflorescences) of two *C. sativa* cultivars from Komga, South Africa, for their essential oils, and antioxidant and anti-inflammatory activities. Eighteen (18) major constituents were identified to be present in fresh and dried flower oils of both cultivars. The dried flower of the Cherrywine cultivar (DCFO) afforded a higher oil yield (1.99 *w*/*w*), while the EOs from the Lifter cultivar contained more chemical constituents (51). Notably, the EOs from both cultivars were cannabinoid-free and contained predominantly oxygenated sesquiterpenes. The EO from the fresh Lifter flower oil (LFO) showed moderate antioxidant activity compared with the other flower oils, based on its considerable DPPH radical scavenging effects. The activity of the LFO may be attributed to its overall chemical composition with the exclusive presence of cis-β-ocimene, fenchol exo-, pinene hydrate, cis-p-mentha-2,8-dien-1-ol, α-terpineol, bicyclogermacrene, nerolidol, ledol, and caryophylla-4 (12),8(13)-dien-5-α-ol. The flower EO samples exhibited potential anti-inflammatory activity in vitro, with potency indicated by their IC_50_ values; however, variability in the inhibitory responses limits the ranking of the samples. Consistent results from the standard control replicates in the antioxidant and anti-inflammatory assays indicate that the method was reliable, and the observed variations among the tested EO samples are unlikely to be due to methodological inconsistencies and are probably due to sample composition and possible storage-related effects. In silico studies suggest β-bisabolene and α-curcumene as potential molecular targets for the observed bioactivity, on the basis of their high binding energy scores with the NOX2 and NIK proteins implicated in ROS and inflammation, respectively. This study has therefore highlighted the considerable antioxidant and anti-inflammatory activity of *C. sativa* flower oils and their potential for managing oxidative stress-related conditions. However, a limitation of the study is that the two cultivars were grown under different environmental conditions; thus, the observed differences in chemical profiles may reflect both genetic variation and environmental effects. Consequently, the present findings should be interpreted with caution as a comparison of essential oil profiles under the respective cultivation conditions, given the limited sample size (*n* = 3), and further studies with increased replication are recommended. Cultivation of both cultivars under the same and controlled environmental conditions would be necessary to distinguish genetic effects from environmental influences. Future directions may also include structural characterization of the major compounds, mechanistic and dose–response confirmation, evaluating the toxicity, including MTT cytotoxicity, total ROS analysis, pharmacokinetic properties and in vivo activity of the oils against oxidative damage to DNA, lipids and proteins.

## Figures and Tables

**Table 1:** Percentage yield and physicochemical analysis of floral EOs of two *C. sativa* cultivars.

Sample Codes	Total Weight of Fresh Plant (g)	Net Weight of Oils (g)	% Yield (*w*/*w*)	Color	Odor/Scent
LFO	189.3	1.22	0.64	Bright yellow	Sweet
CFO	361.00	0.97	0.27	Pale yellow	Mildly sweet
DLFO	189.3	1.72	0.91	Yellow	Mildly floral
DCFO	100	1.99	1.99	Transparent	Mildly sweet

Fresh (LFO; CFO) and dried (DLFO; DCFO) flower oils of two *C. sativa* cultivars.

**Table 2:** Chemical composition of EOs from fresh and dried flowers of two cultivars of *C. sativa*.

				RI Lit.	Percent Composition of Plant Parts (%)			
RT (Min)	Chemical Components Identified ^a^	CAS No.	RI Cal. ^b^		LFO ^c^	DLFO ^d^	CFO ^c^	DCFO ^d^
8.175	1-Vinyl-5,5-dimethyl[2.1.1]bicyclohexane	016626-39-4	919	920	-	0.65	0.10	-
8.815	α-Pinene	000080-56-8	936	936	2.22	5.22	3.17	3.19
9.370	Camphene	000079-92-5	950	950	-	0.54	-	-
10.498	Levo-β-pinene	018172-67-3	980	992	12.35	3.11	13.21	1.37
11.245	β-Pinene	000127-91-3	1000	976	-	-	-	13.96
11.272	β-Terpinene	000099-84-3	1000	1020	-	19.23	-	-
12.544	D-limonene	005989-27-5	1033	1030	2.88	5.48	1.76	1.40
12.936	Trans-β-ocimene	003779-61-1	1044	1048	-	-	-	0.33
13.301	β-Ocimene	013877-91-3	1053	1050	-	0.90	2.72	-
13.318	cis-β-Ocimene, (Z)	003338-55-4	1054	1053	0.22	-	-	4.37
14.611	L-fenchone	007787-20-4	1087	**	-	0.33	-	-
14.996	Rosefuran	015186-51-3	1098	1093	-	-	0.07	
15.122	Linalool	000078-70-6	1101	1103	1.49	4.28	1.12	2.18
15.489	Fenchol exo-	022627-95-8	1110	1117	0.37	-	-	0.16
15.503	Fenchol	001632-73-1	1111	1123	-	1.27	0.15	-
15.738	Pinene hydrate	000473-54-1	1117	1145	0.27	-	-	-
15.745	trans-2-Pinanol	004948-29-2	1117	1118	-	0.79	0.15	-
16.213	cis-p-Mentha-2,8-dien-1-ol	003886-78-0	1129	1131	0.16	-	-	-
16.477	Myroxide	028977-57-3	1136	1138	-	-	0.18	-
17.191	Endo-borneol	000507-70-0	1155	1156	0.21	0.60	-	-
17.984	α-Terpineol	000098-55-5	1176	1189	0.75	-	-	0.19
17.994	Terpineol	1000411-59-6	1176	**	-	1.38	0.14	-
18.547	2,6-Dimethyl-3,5,7-octatriene-2-ol, E,E-	1000141-11-8	1190	1208	-	-	0.19	-
23.183	Ylangene	014912-44-8	1400	1367	0.16	0.20	-	-
23.305	α-Copaene	1000360-33-0	1377	1370	-	-	0.07	-
23.556	n-Hexyl hexanoate	006378-65-0	1378	1386	0.63	0.33	-	-
24.128	(Z,Z)-α-farnesene	1000293-03-1	1411	1462	-	-	0.63	-
24.134	Isocaryophyllene	000118-65-0	1411	1406	-	-	-	0.63
24.544	Caryophyllene	000087-44-5	1428	1423	10.71	11.33	19.59	19.96
24.723	(E)-β-farnesene	018794-84-8	1435	1442	-	-	0.12	-
24.883	trans-α-Bergamotene	013474-59-4	1441	1437	0.68	0.70	2.37	2.42
24.967	α-Guaiene	003691-12-1	1445	1439	-	-	4.82	5.62
25.371	Humulene	006753-98-6	1461	1457	6.03	5.88	9.77	9.62
25.518	Alloaromadendrene	025246-27-9	1467	1462	0.95	1.11	-	-
25.732	4,5-Di-epi-aristolochene	1000374-17-1	1476	1471	-	-	0.11	-
25.900	γ-Muurolene	030021-74-0	1483	1480	-	0.27	-	-
25.989	α-Amorphene	020085-19-2	1487	1485	0.26	0.25	-	-
26.035	α-Curcumene	000644-30-4	1489	1480	-	-	0.22	0.32
26.092	Naphthalene,1,2,3,4,4a,5,6,7-octahydro-4a,8-dimethyl-2-(1-methylethenyl)-	103827-22-1	1491	1492	0.67	0.58	0.87	0.90
26.166	(+)-β-Selinene	017066-67-0	1494	1492	1.36	1.20	2.40	2.51
26.323	Valencene	004630-07-3	1500	1503	3.60	0.82	-	-
26.379	α-Selinene	000473-13-2	1503	1493	-	-	0.15	0.14
26.385	4,11-Selinadiene	1000192-43-5	1503	1473	-	-	2.25	-
26.472	Guaia-9,11-diene	1000374-19-8	1507	1521	-	-	0.21	-
26.665	β-Bisabolene	000495-61-4	1514	1514	1.25	1.52	-	-
26.675	δ-Guaiene	003691-11-0	1515	1509	-	-	9.72	10.91
26.824	γ-Cadinene	039029-41-9	1521	1513	0.22	0.22	-	-
26.919	β-Cyclogermacrene	024703-35-3	1525	1505	-	0.55	-	-
26.920	Bicyclogermacrene	067650-90-2	1525	1505	0.68	-	-	
27.035	β-Sesquiphellandrene	020307-83-9	1530	1525	-	-	0.54	0.67
27.345	Naphthalene, decahydro-4a-methyl-1-methylene-7-(1-methylethylidene)-, trans-	058893-88-2	1542	1544	-	2.22	-	-
27.505	Selina-3,7(11)-diene	006813-21-4	1549	1546	4.23	3.47	0.15	-
27.866	β-Germacrene	015423-57-1	1564	1558	0.52	0.19	-	-
27.970	Nerolidol	007212-44-4	1568	1556	1.03	-	-	
27.989	Nerolidol	000142-50-7	1569	1570	-	0.63	4.18	4.97
28.548	Caryophyllene oxide	001139-30-6	1592	1583	7.49	4.32	7.47	5.59
28.827	Guaiol	000489-86-1	1603	1600	2.60	1.54	0.17	-
28.969	Ledol	000577-27-5	1609	1608	0.32	-	-	-
29.117	Humulene-1,2-epoxide	019888-34-7	1615	1603	3.32	1.67	1.75	1.23
29.216	Patchoulane	025491-20-7	1619	1618	-	-	-	0.12
29.352	7-Epi-γ-eudesmol	117066-77-0	1624	1660	3.81	1.96	0.17	-
29.506	Isoaromadendrene epoxide	1000159-36-6	1631	1590	-	0.33	-	-
29.635	Caryophylla-4(12),8(13)-dien-5-α-ol	019431-79-9	1636	1637	0.56	-	-	-
29.698	Caryophylla-4(12),8(13)-dien-5-β-ol	019431-80-2	1639	1644	-	-	0.27	0.18
30.045	β-Eudesmol	000473-15-4	1653	1652	1.94	0.75	-	-
30.108	α-Eudesmol	000473-16-5	1655	1643	2.68	1.17	-	-
30.405	Bulnesol	022451-73-6	1668	1666	1.88	0.80	-	-
30.772	α-Bisabolol	000515-69-5	1683	1685	5.74	2.54	-	-
31.046	Juniper camphor	000473-04-1	1694	1700	0.48	0.21	-	-
31.300	Rotundone	018374-76-0	1745	1722	-	-	0.11	
36.253	m-Camphorene	020016-73-3	1958	1944	-	0.23	0.09	-
	Total compounds identified				85.80	90.77	91.50	92.95
	Total monoterpenes (%)				20.92	43.78	22.97	27.16
	Total sesquiterpenes (%)				64.88	46.43	68.44	65.79
	Others (%)				-	0.56	0.09	-

^a^ Compounds are listed in order of their retention time (RT). ^b^ RI (retention index) measured relative to n-alkanes (C9–C36) using a ZB-5MS capillary column. ^c^ Essential oil of fresh plant parts (Lifter and Cherrywine). ^d^ Essential oil of dried plant parts (Lifter and Cherrywine). ** No literature for RI. Bold values refer to the main constituents of the EOs.

**Table 3:** In vitro antioxidant activities (IC_50_) of flower oils of two *C. sativa* cultivars.

Test Samples	IC_50_ ± SD (µg/mL)
	DPPH
LFO	69.50 ± 4.05 ***
DLFO	ND
CFO	ND
DCFO	ND
Ascorbic acid	11.28 ± 0.20

Data are expressed as the mean ± standard deviation (*n* = 3). *** (*p* ≤ 0.001) of fresh Lifter (LFO), when compared with the standard L-ascorbic acid. ND = not determined in dried Lifter (DLFO), fresh Cherrywine (CFO) and dried Cherrywine (DCFO) flower oils.

**Table 4:** In vitro anti-inflammatory activities (IC_50_) of flower oils of two *C. sativa* cultivars.

Test Samples	IC_50_ ± SD (µg/mL)
	DPPH
LFO	16.12 ± 10.01
DLFO	18.61 ± 2.27
CFO	20.36 ± 3.88
DCFO	78.66 ± 6.31
Diclofenac	9.25 ± 1.09

Data are expressed as the mean ± standard deviation (*n* = 3). Fresh Lifter flower oil (LFO), dried Lifter flower oil (DLFO), fresh Cherrywine flower oil (CFO) and dried Cherrywine flower oil (DCFO).

**Table 5:** Binding energy scores of the essential oil compounds found exclusively in the dried and fresh flowers of the *C. sativa* Cherrywine cultivar with NOX2 protein.

S/No.	Compound Name	Binding Affinity (Kcal/mol)
1	β-Pinene	−6.5
2	trans-β-Ocimene	−5.5
3	β-cis-Ocimene	−5.8
4	Rosefuran	−7.1
5	Myroxide	−6.4
6	2,6-Dimethyl-3,5,7-octatriene-2-ol, E,E-	−5.7
7	α-Copaene	−6.2
8	(Z,Z)-α-farnesene	−6.4
9	Isocaryophyllene	−6.1
10	(E)-β-farnesene	−5.9
11	α-Guaiene	−4.2
12	4,5-Di-epi-aristolochene	−5.1
13	α-Curcumene	−7.5
14	4,11-Selinadiene	−5.3
15	Guaia-9,11-diene	−6.1
16	β-Sesquiphellandrene	−6.5
17	Patchoulane	−4.9
18	Caryophylla-4(12),8(13)-dien-5-β-ol;	−5.0
19	α-Selinene	−6.3
20	δ-Guaiene	−6.6
21	Rotundone	−5.7

**Table 6:** Binding energy scores of the essential oil compounds found exclusively in the dried and fresh flowers of the *C. sativa* Lifter cultivar with NIK protein.

S/No.	Compound Name	Binding Affinity (Kcal/mol)
1	Camphene	−5.3
2	β-Terpinene	−6.1
3	L-fenchone	−6.5
4	Pinene hydrate	−4.9
5	cis-p-Mentha-2,8-dien-1-ol	−5.0
6	Endo-borneol	−6.3
7	Ylangene	−6.6
8	n-Hexyl hexanoate	−4.2
9	Alloaromadendrene	−5.1
10	γ-Muurolene	−4.9
11	α-Amorphene	−4.4
12	Valencene	−4.0
13	β-Bisabolene	−7.7
14	γ-Cadinene	−5.8
15	β-Cyclogermacrane	−7.1
16	Bicyclogermacrene	−6.4
17	Naphthalene, decahydro-4a-methyl-1-methylene-7-(1-methylethylidene)-, trans-	−5.7
18	β-Germacrene	−6.2
19	Nerolidol (CAS No. = 007212-44-4)	−6.4
20	Ledol	−6.1
21	Isoaromadendrene epoxide	−5.9
22	Caryophylla-4(12),8(13)-dien-5-α-ol	−4.2
23	β-Eudesmol	−5.1
24	α-Eudesmol	−4.9
25	Bulnesol	−4.4
26	α-Bisabolol	−5.1
27	Juniper camphor	−5.2

## Data Availability

The data are within the manuscript.

## Supplementary Materials

The following supporting information can be downloaded at: https://www.mdpi.com/article/10.3390/molecules31111814/s1, Figure S1: GC-MS Total Ion Chromatograms of: (**a**) Fresh Lifter flower oil (LFO); (**b**) Dried Lifter flower oil (DLFO); (**c**) Fresh Cherrywine flower oil (CFO); (**d**) Dried Cherrywine flower oil (DCFO).

## Abbreviations

The following abbreviations for the amino acids found in proteins, as well as other abbreviations, are used in this manuscript.

Ile	Isoleucine
Leu	Leucine
Phe	Phenylalanine
Arg	Arginine
His	Histidine
Ser	Serine
Trp	Tryptophan
Val	Valine
Tyr	Tyrosine
Lys	Lysine
Ala	Alanine
Thr	Threonine
Gly	Glycine
Glu	Glutamic acid
Gln	Glutamine
Asp	Aspartic acid
Asn	Asparagine
Cys	Cysteine
Met	Methionine
CAT	Catalase (enzyme)
GSH	Reduced glutathione
IC_50_	Inhibitory concentration
ABSSample	Absorbance value of samples
ABSControl	Absorbance value of control
DNA	Deoxyribonucleic acid
NOX2	NADPH oxidase 2
NADPH	Nicotinamide adenine dinucleotide phosphate (reduced form)
NF-κβ	Nuclear factor kappa B
NIK	NF-κB-inducing kinase
EAD	Egg albumin denaturation
VOCs	Volatile organic compounds
MVA	Mevalonic acid
MEP	Methylerythritol
EOs	Essential oils
ROS	Reactive oxygen species
MTT cytotoxicity	3-(4,5-Dimethylthiazol-2-yl)-2,5-diphenyltetrazolium bromide

## References

[1] Lo M.M., Benfodda Z., Molinié R., Meffre P. (2024). Volatile organic compounds emitted by flowers: Ecological roles, production by plants, extraction, and identification. Plants.

[2] Mofikoya O.O. (2022). Chemical Fingerprinting of Conifer Needle Extracts by Ultrahigh Resolution Mass Spectrometry.

[3] de Araújo D.A.M., Freitas C., Cruz J.S. (2011). Essential oils components as a new path to understand ion channel molecular pharmacology. Life Sci..

[4] Mukaila Y.O., Pfukwa T.M., Fawole O.A. (2025). Essential oils from South African indigenous plants: Extraction techniques, phytochemistry, biological activities and applications. South Afr. J. Bot..

[5] Sadgrove N.J., Padilla-González G.F., Phumthum M. (2022). Fundamental chemistry of essential oils and volatile organic compounds, methods of analysis and authentication. Plants.

[6] Chamorro E.R., Zambón S.N., Morales W.G., Sequeira A.F., Velasco G.A. (2012). Study of the chemical composition of essential oils by gas chromatography. Gas Chromatogr. Plant Sci. Wine Technol. Toxicol. Some Specif. Appl..

[7] Zuzarte M., Salgueiro L. (2015). Essential Oils Chemistry. Bioactive Essential Oils and Cancer.

[8] El Asbahani A., Miladi K., Badri W., Sala M., Aït Addi E.H., Casabianca H., El Mousadik A., Hartmann D., Jilale A., Renaud F.N.R. (2015). Essential oils: From extraction to encapsulation. Int. J. Pharm..

[9] Muresan S.M.C., Dreanca A., Repciuc C., Dejescu C., Rotar O., Pop R.A., Pantea S., Pall E., Ciotlaus I., Sarosi C. (2023). Dental hydrogels with essential oils with potential activity in periodontitis. Appl. Sci..

[10] Chaieb K., Hajlaoui H., Zmantar T., Kahla-Nakbi A.B., Rouabhia M., Mahdouani K., Bakhrouf A. (2007). The chemical composition and biological activity of clove essential oil, *Eugenia caryophyllata* (*Syzigium aromaticum* L. Myrtaceae): A short review. Phytother. Res. Int. J. Devoted Pharmacol. Toxicol. Eval. Nat. Prod. Deriv..

[11] Mancianti F., Ebani V.V. (2020). Biological activity of essential oils. Molecules.

[12] Raut J.S., Karuppayil S.M. (2014). A status review on the medicinal properties of essential oils. Ind. Crops Prod..

[13] Odieka A.E., Obuzor G.U., Oyedeji O.O., Gondwe M., Hosu Y.S., Oyedeji A.O. (2022). The medicinal natural products of *Cannabis sativa* Linn.: A review. Molecules.

[14] Tajabadi F., Nohooji M.G., Hajiaghaee R. (2024). Identification and resolving of trace and co-eluted components of Lamium amplexicaule essential oil using two chemometric methods-assisted GC-MS. Trends Pharm. Sci..

[15] Kalogiouri N.P., Manousi N., Rosenberg E., Zachariadis G.A., Samanidou V.F. (2021). Advances in the chromatographic separation and determination of bioactive compounds for assessing the nutrient profile of nuts. Curr. Anal. Chem..

[16] Janta P., Vimolmangkang S. (2024). Chemical profiling and clustering of various dried cannabis flowers revealed by volatilomics and chemometric processing. J. Cannabis Res..

[17] Moreno-Chamba B., Salazar-Bermeo J., Hosseinian F., Martin-Bermudo F., Aguado M., De la Torre R., Martínez-Madrid M.C., Valero M., Martí N., Saura D. (2024). Aromatic and cannabinoid profiles of Cannabis inflorescences and seed oils: A comprehensive approach for variety characterization. Ind. Crops Prod..

[18] Lu Y., Young S., Linder E., Whipker B., Suchoff D. (2022). Hyperspectral imaging with machine learning to differentiate cultivars, growth stages, flowers, and leaves of industrial hemp (*Cannabis sativa* L.). Front. Plant Sci..

[19] Šovljanski O., Aćimović M., Sikora V., Koren A., Saveljić A., Tomić A., Tešević V. (2024). Exploring (Un) Covered Potentials of Industrial Hemp (*Cannabis sativa* L.) Essential Oil and Hydrolate: From Chemical Characterization to Biological Activities. Nat. Prod. Commun..

[20] Di Sotto A., Gullì M., Acquaviva A., Tacchini M., Di Simone S.C., Chiavaroli A., Recinella L., Leone S., Brunetti L., Orlando G. (2022). Phytochemical and pharmacological profiles of the essential oil from the inflorescences of the *Cannabis sativa* L.. Ind. Crops Prod..

[21] Marques S.D.P.P.M., Pinheiro R.O., Nascimento R.A.D., Andrade E.H.D.A., Faria L.J.G.D. (2023). Effects of Harvest Time and Hydrodistillation Time on Yield, Composition, and Antioxidant Activity of Mint Essential Oil. Molecules.

[22] Qamar S., Torres Y.J., Parekh H.S., Falconer J.R. (2021). Extraction of medicinal cannabinoids through supercritical carbon dioxide technologies: A review. J. Chromatogr. B.

[23] Kaminski K.P., Hoeng J., Lach-Falcone K., Goffman F., Schlage W.K., Latino D. (2025). Exploring Aroma and Flavor Diversity in *Cannabis sativa* L.—A Review of Scientific Developments and Applications. Molecules.

[24] Fiorentino N., Formisano C., Delfine S., Chianese G. (2024). Editorial: Environmental and Agronomic Factors Affecting the Chemical Composition and Biological Activity of Cannabis Extracts. Front. Plant Sci..

[25] Radwan M.M., Chandra S., Gul S., Elsohly M.A. (2021). Cannabinoids, Phenolics, Terpenes and Alkaloids of Cannabis. Molecules.

[26] Sommano S.R., Chittasupho C., Ruksiriwanich W., Jantrawut P. (2020). The Cannabis Terpenes. Molecules.

[27] Pino S., Espinoza L., Jara-Gutiérrez C., Villena J., Olea A.F., Díaz K. (2023). Study of cannabis oils obtained from three varieties of *C. sativa* and by two different extraction methods: Phytochemical characterization and biological activities. Plants.

[28] Kwaśnica A., Pachura N., Carbonell-Barrachina Á.A., Issa-Issa H., Szumny D., Figiel A., Masztalerz K., Klemens M., Szumny A. (2023). Effect of drying methods on chemical and sensory properties of *Cannabis sativa* leaves. Molecules.

[29] Birenboim M., Kengisbuch D., Chalupowicz D., Maurer D., Barel S., Chen Y., Fallik E., Paz-Kagan T., Shimshoni J.A. (2022). Use of near-infrared spectroscopy for the classification of medicinal cannabis cultivars and the prediction of their cannabinoid and terpene contents. Phytochemistry.

[30] García-Valverde M.T., de Medina V.S., Codesido V., Hidalgo-García J., Ferreiro-Vera C. (2020). Exploring the Mysteries of Cannabis Through Gas Chromatography. Recent Advances in Gas Chromatography.

[31] Gilbert A.N., DiVerdi J.A. (2018). Consumer perceptions of strain differences in Cannabis aroma. PLoS ONE.

[32] Vigil J.M., Stith S.S., Brockelman F., Keeling K., Hall B. (2023). Systematic combinations of major cannabinoid and terpene contents in Cannabis flower and patient outcomes: A proof-of-concept assessment of the Vigil Index of Cannabis Chemovars. J. Cannabis Res..

[33] Hanuš L.O., Hod Y. (2020). Terpenes/terpenoids in cannabis: Are they important?. Med. Cannabis Cannabinoids.

[34] Rajapaksha H., Fernando M., Nelumdeniya N., Bandara A., Silva A. (2024). Evaluation of in vitro anti-inflammatory activity and In-silico pharmacokinetics and molecular docking study of Horsfieldia iryaghedhi. J. Phytopharm..

[35] Khan M.S.A., Ahmad I. (2019). Herbal Medicine: Current Trends and Future Prospects. New Look to Phytomedicine.

[36] El-Mernissi R., El Menyiy N., Zouhri A., El-Mernissi Y., Diai F., Siddique F., Dabiellil F., Almaary K.S., Amhamdi H., Abboussi O. (2024). Phytochemical profiling and bioactivity evaluation of CBD-and THC-enriched *Cannabis sativa* extracts: In vitro and in silico investigation of antioxidant and anti-inflammatory effects. Open Chem..

[37] Sankarikutty B., Narayanan C. (2003). Essential Oils: Isolation and Production. Encyclopaedia of Food Science, Food Technology and Nutrition.

[38] Raeber J., Bajor B., Poetzsch M., Steuer C. (2025). Comprehensive analysis of chemical and enantiomeric stability of terpenes in Cannabis sativa L. flowers. Phytochem. Anal..

[39] Kwaśnica A., Pachura N., Masztalerz K., Figiel A., Zimmer A., Kupczyński R., Wujcikowska K., Carbonell-Barrachina A.A., Szumny A., Różański H. (2020). Volatile composition and sensory properties as quality attributes of fresh and dried hemp flowers (*Cannabis sativa* L.). Foods.

[40] Ahn J.H., Lee T.W., Kim K.H., Byun H., Ryu B., Lee K.T., Jang D.S., Choi J.H. (2015). 6-Acetoxy cyperene, a patchoulane-type sesquiterpene isolated from *Cyperus rotundus* rhizomes induces caspase-dependent apoptosis in human ovarian cancer cells. Phytother. Res..

[41] Boulebd H. (2021). Are thymol, rosefuran, terpinolene and umbelliferone good scavengers of peroxyl radicals?. Phytochemistry.

[42] Malaspina P., Polito F., Mainetti A., Khedhri S., De Feo V., Cornara L. (2025). Exploring Chemical Variability in the Essen-tial Oil of *Artemisia absinthium* L. in Relation to Different Phenological Stages and Geographical Location. Chem. Biodivers..

[43] Hossain F., Ashraful A.B.M., Hossain M.J., Rashid M.A., Alam A.K. (2025). The Pharmacological Prospects of Curcuma: A Review of Its Antimicrobial, Antioxidant, and Anticancer Properties. Dhaka Univ. J. Pharm. Sci..

[44] Amil M.A., Rahman S.N.S.A., Yap L.F., Razak F.A., Bakri M.M., Salem L.S., Lim X.Y., Reduan N.A., Sim K.S. (2024). Anti-microbial and antiproliferative effects of zingiberaceae oils: A natural solution for oral health. Chem. Biodivers..

[45] Chaturvedi T., Singh S., Nishad I., Kumar A., Tiwari N., Tandon S., Saikia D., Verma R.S. (2021). Chemical composition and antimicrobial activity of the essential oil of senescent leaves of guava (*Psidium guajava* L.). Nat. Prod. Res..

[46] Silva R.C., Costa J.S., Figueiredo R.O., Setzer W.N., Silva J.K.R., Maia J.G.S., Figueiredo P.L.B. (2021). Monoterpenes and Sesquiterpenes of Essential Oils from Psidium Species and Their Biological Properties. Molecules.

[47] Pieracci Y., Ascrizzi R., Terreni V., Pistelli L., Flamini G., Bassolino L., Fulvio F., Montanari M., Paris R. (2021). Essential oil of *Cannabis sativa* L: Comparison of yield and chemical composition of 11 hemp genotypes. Molecules.

[48] Calva J., Silva M., Morocho V. (2023). Composition and anti-acetylcholinesterase properties of the essential oil of the Ecuadorian endemic species *Eugenia valvata* McVaugh. Molecules.

[49] Tran G.B., Pham T.V., Tuan Le A., Nguyen N.H., Vo N.H.H., Do B.H. (2024). Chemical composition and the anti-inflammatory effect of volatile compounds from Anaxagorea luzonensis A. Gray. Z. Für Naturforschung C.

[50] Christodoulou M.C., Orellana Palacios J.C., Hesami G., Jafarzadeh S., Lorenzo J.M., Domínguez R., Moreno A., Hadidi M. (2022). Spectrophotometric methods for measurement of antioxidant activity in food and pharmaceuticals. Antioxidants.

[51] Judžentienė A., Garjonytė R., Būdienė J. (2023). Phytochemical Composition and Antioxidant Activity of Various Extracts of Fibre Hemp (*Cannabis sativa* L.) Cultivated in Lithuania. Molecules.

[52] Dahiya M., Kumar A., Yadav M., Chauhan S. (2025). β-pinene ameliorates ICV-STZ induced Alzheimer′s pathology via antioxidant, anticholinesterase, and mitochondrial protective effects: In-silico and in-vivo studies. Eur. J. Pharmacol..

[53] Tisserand R., Young R. (2014). Essential Oil Safety: A Guide for Health Care Professionals.

[54] Chaiya P., Senarat S., Phaechamud T., Narakornwit W. (2022). In vitro anti-inflammatory activity using thermally inhibiting protein denaturation of egg albumin and antimicrobial activities of some organic solvents. Mat. Today Proc..

[55] Najafian S. (2025). Preservation of essential oil quality in endangered *Ziziphora tenuior* L. under different storage conditions. Sci. Rep..

[56] Li Y., Li Y., Fang T., Feng X., Gao D. (2025). Impact of processing methods and storage on the chemical composition of *Artemisia argyi* essential oil. Ind. Crops Prod..

[57] (2019). Standard Practice for Laboratory Test Method Validation and Method Development.

[58] Naz S., Hanif M.A., Bhatti H.N., Ansari T.M. (2017). Impact of supercritical fluid extraction and traditional distillation on the isolation of aromatic compounds from *Cannabis indica* and *Cannabis sativa*. J. Essent. Oil Bear. Plants.

[59] Oyedeji O., Yani V., Afolayan A. (2005). Chemical composition of the essential oil from *Arctotis arctotoides* (LF) O. Hoffm. (syn. *Vendium arctotoides* Less.). Flavour Fragr. J..

[60] Trujillo-Echeverria L., Lara Fiallos M.V., de la Vega Quintero J.C., Espín Valladares R., Guardado Yordi E., Radice M., Pérez Martínez A. (2020). Technical-economic analysis of the solvent-method optimization of *Origanum vulgare* essential oil extraction based on technical and quality criteria. SN Appl. Sci..

[61] Babushok V., Linstrom P., Zenkevich I. (2011). Retention indices for frequently reported compounds of plant essential oils. J. Phys. Chem. Ref. Data.

[62] Adams R.P., Beauchamp P.S., Dev V., Dutz S.M. (2007). New natural products isolated from one-seeded *Juniperus* of the Southwestern United States: Isolation and occurrence of 2-ethenyl-3-methyl phenol and its derivatives. J. Essent. Oil Res..

[63] Costa O.B.D., Del Menezzi C.H.S., Benedito L.E.C., Resck I.S., Vieira R.F., Ribeiro Bizzo H. (2014). Essential oil constituents and yields from leaves of *Blepharocalyx salicifolius* (Kunt) O. Berg and *Myracrodruon urundeuva* (Allemão) collected during daytime. Int. J. For. Res..

[64] Oriola A.O., Miya G.M., Singh M., Oyedeji A.O. (2023). Flavonol glycosides from *Eugenia uniflora* leaves and their in vitro cytotoxicity, antioxidant and anti-inflammatory activities. Sci. Pharm..

[65] Ameena M., Arumugham M., Ramalingam K., Rajeshkumar S., Shanmugam R. (2023). Evaluation of the anti-inflammatory, antimicrobial, antioxidant, and cytotoxic effects of chitosan thiocolchicoside-lauric acid nanogel. Cureus.

[66] Noreng S., Ota N., Sun Y., Ho H., Johnson M., Arthur C.P., Schneider K., Lehoux I., Davies C.W., Mortara K. (2022). Structure of the core human NADPH oxidase NOX2. Nat. Commun..

[67] Liu J., Sudom A., Min X., Cao Z., Gao X., Ayres M., Lee F., Cao P., Johnstone S., Plotnikova O. (2012). Structure of the nuclear factor κB-inducing kinase (NIK) kinase domain reveals a constitutively active conformation. J. Biol. Chem..

[68] O’Boyle N.M., Banck M., James C.A., Morley C., Vandermeersch T., Hutchison G.R. (2011). Open Babel: An open chemical toolbox. J. Cheminf..

[69] Schüttelkopf A.W., Van Aalten D.M. (2004). PRODRG: A tool for high-throughput crystallography of protein–ligand complexes. Biol. Crystallogr..

[70] Kar P., Sharma N.R., Singh B., Sen A., Roy A. (2021). Natural compounds from *Clerodendrum* spp. as possible therapeutic candidates against SARS-CoV-2: An in silico investigation. J. Biomol. Struct. Dyn..

[71] Kar P., Saleh-E-In M.M., Jaishee N., Anandraj A., Kormuth E., Vellingiri B., Angione C., Rahman P.K., Pillay S., Sen A. (2022). Computational profiling of natural compounds as promising inhibitors against the spike proteins of SARS-CoV-2 wild-type and the variants of concern, viral cell-entry process, and cytokine storm in COVID-19. J. Cell. Biochem..

