# Supplementary material for: Chemical Characterization and Biological Potential of the Essential Oils from the Flowers of Two Cannabis sativa L. Cultivars from Komga, South Africa
Source: Molecules. 2026 May 25;31(11):1814. doi: 10.3390/molecules31111814 (PMC13257954; doi:10.3390/molecules31111814)
Supplement: Supplementary file 1 [file molecules-31-01814-s001.zip › molecules-4225639-supplementary.pdf]

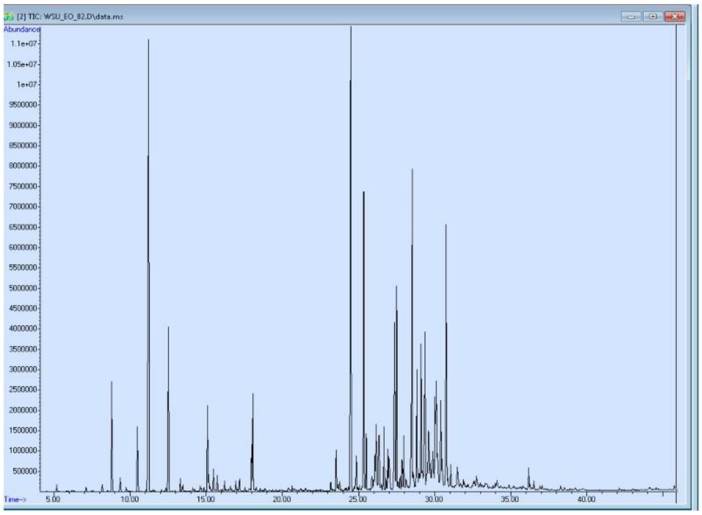

(a)

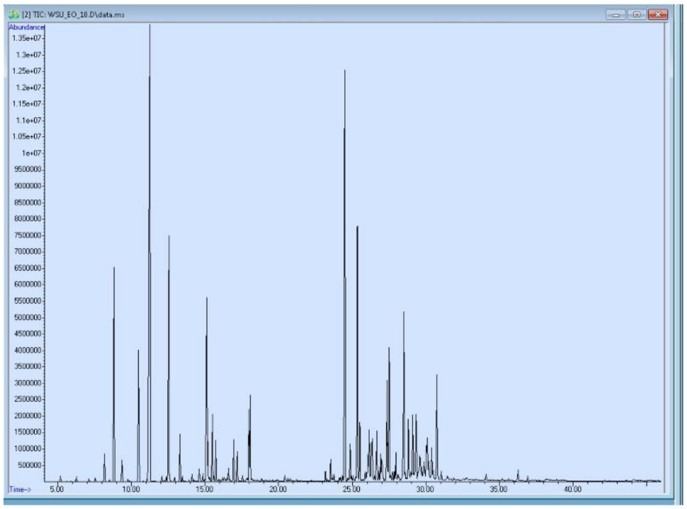

(b)

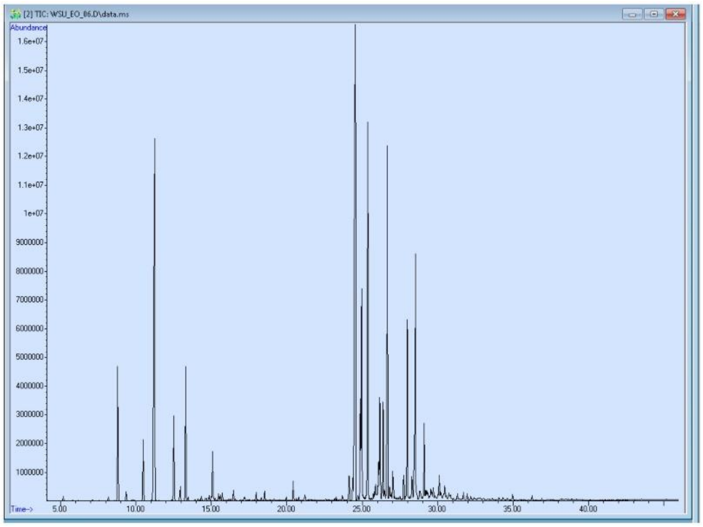

(c)

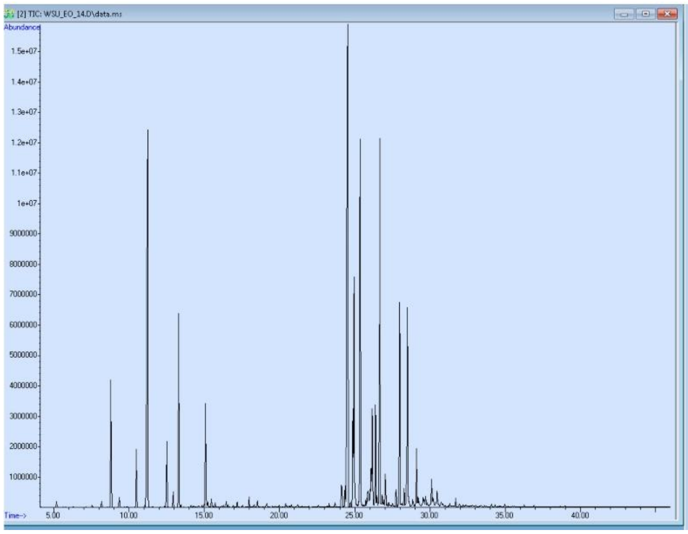

(d)

Figure S1: GC-MS Total Ion Chromatograms of: (**a**) Fresh Lifter flower oil (LFO); (**b**) Dried Lifter flower oil (DLFO); (**c**) Fresh Cherrywine flower oil (CFO); (**d**) Dried Cherrywine flower oil (DCFO).
